# Supplementary figures and images for: Redistribution of fragmented mitochondria ensures symmetric organelle partitioning and faithful chromosome segregation in mitotic mouse zygotes
Source: eLife. 2025 Aug 11;13:RP99936. doi: 10.7554/eLife.99936 (PMC12339001; doi:10.7554/eLife.99936)

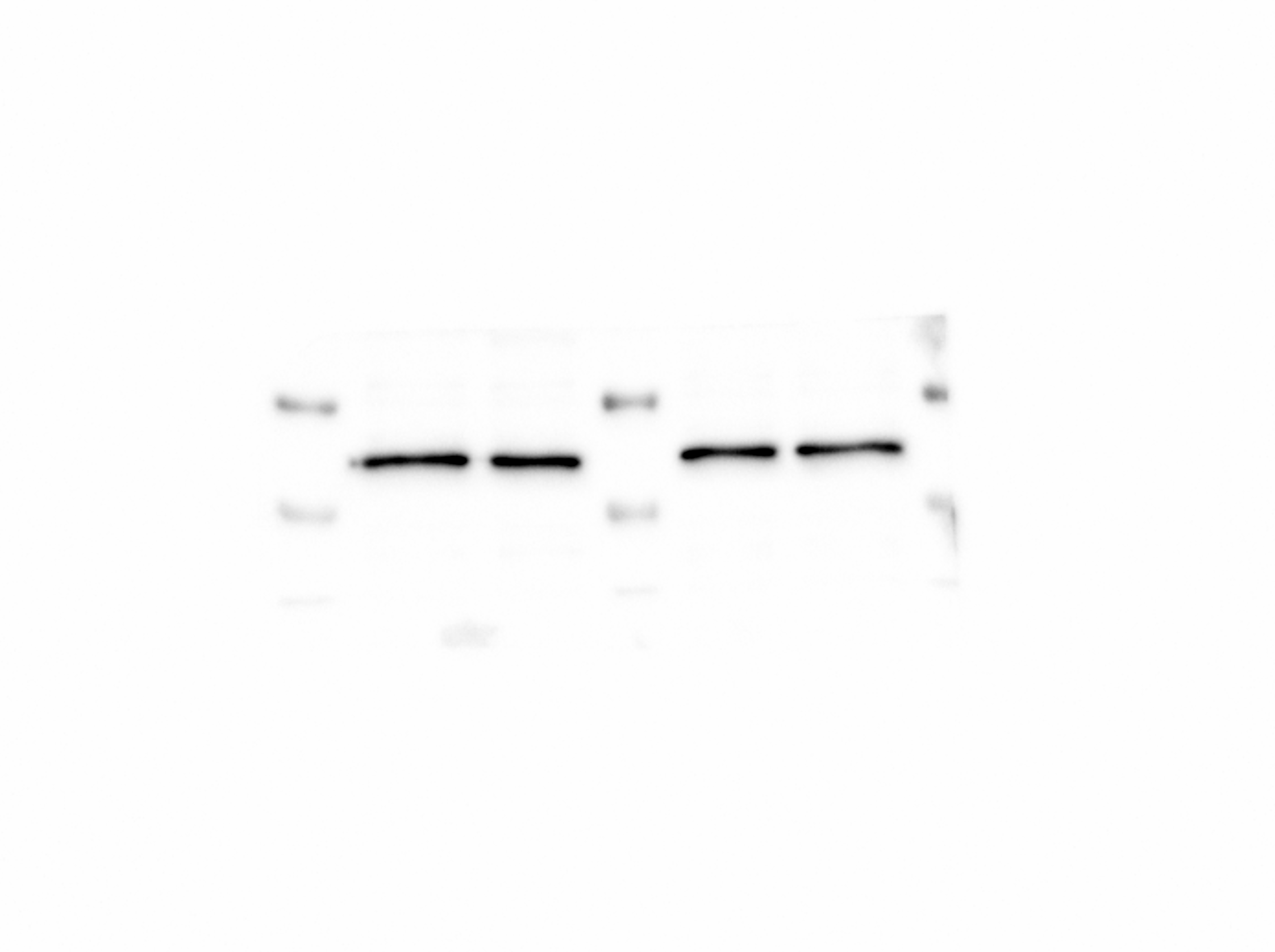

Supplement: Figure 1—source data 1. [file elife-99936-fig1-data1.zip › Figure 1-Source Data 1 (raw)/Actin_user 2023-09-20_18h55m32s_Exposure_1.0sec.tif]

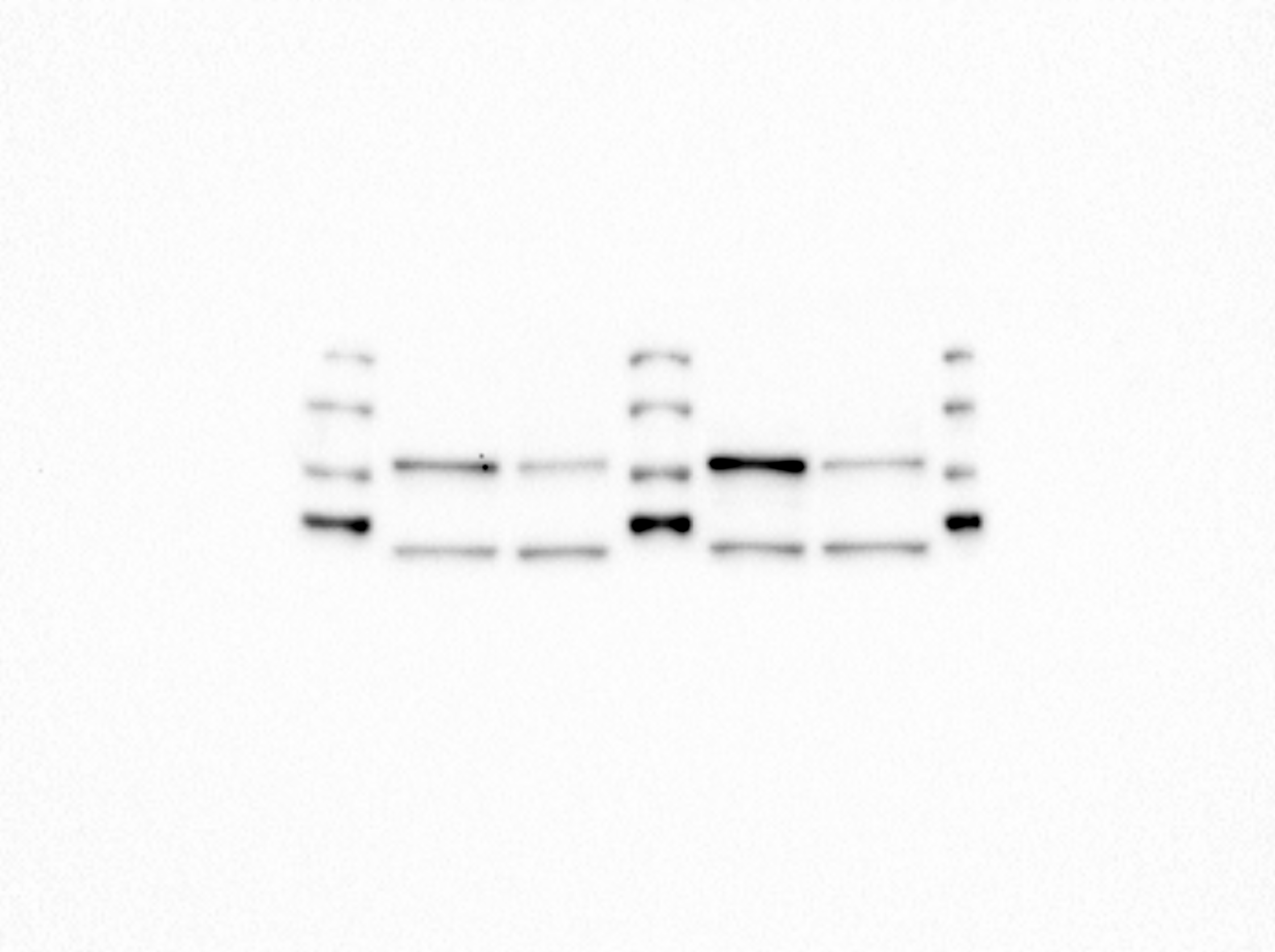

Supplement: Figure 1—source data 1. [file elife-99936-fig1-data1.zip › Figure 1-Source Data 1 (raw)/Myo19_user 2023-09-20_18h28m45s_Exposure_10.0sec.tif]

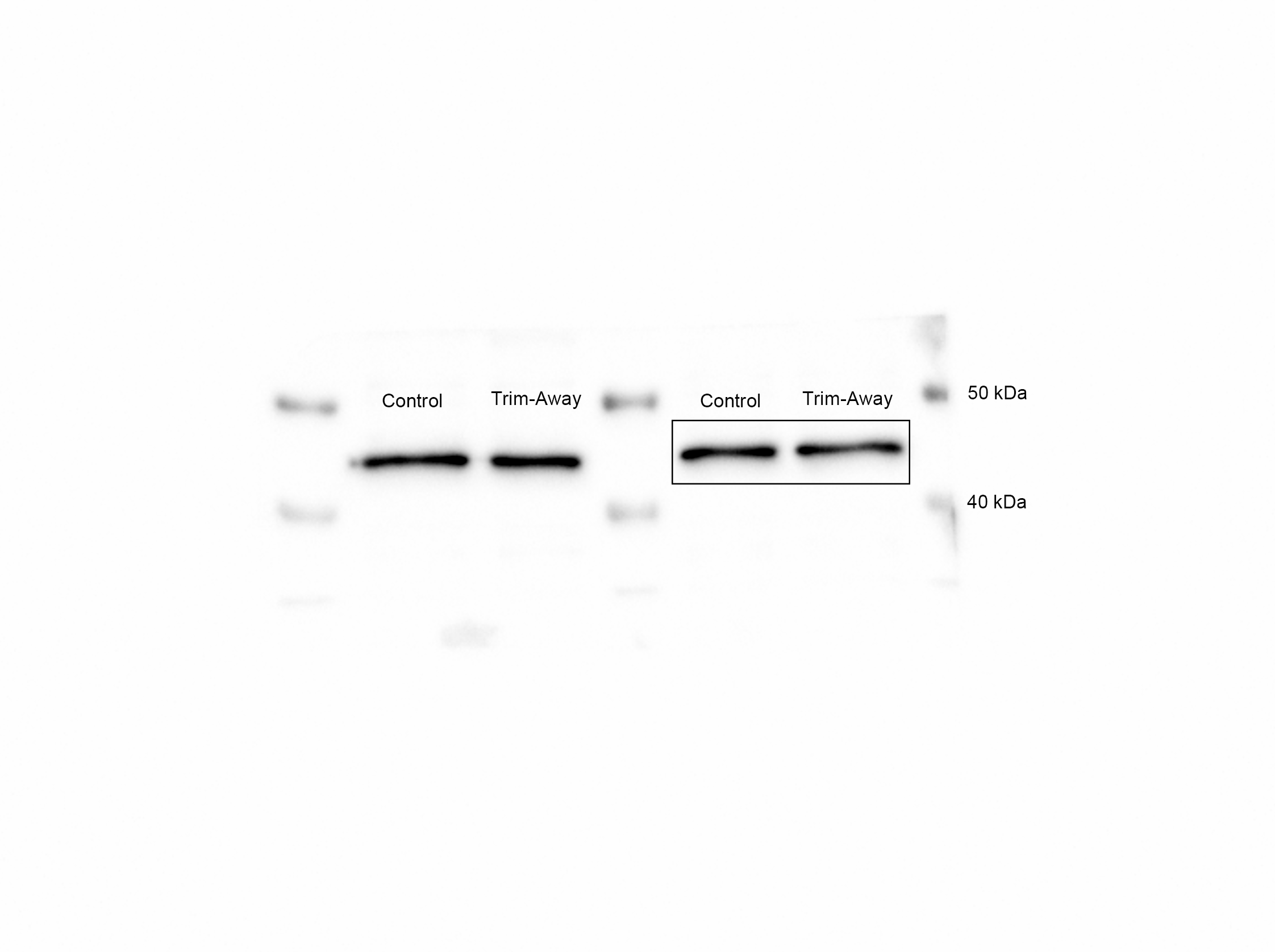

Supplement: Figure 1—source data 2. [file elife-99936-fig1-data2.zip › Figure 1-Source Data 2/Actin Trim Away.tif]

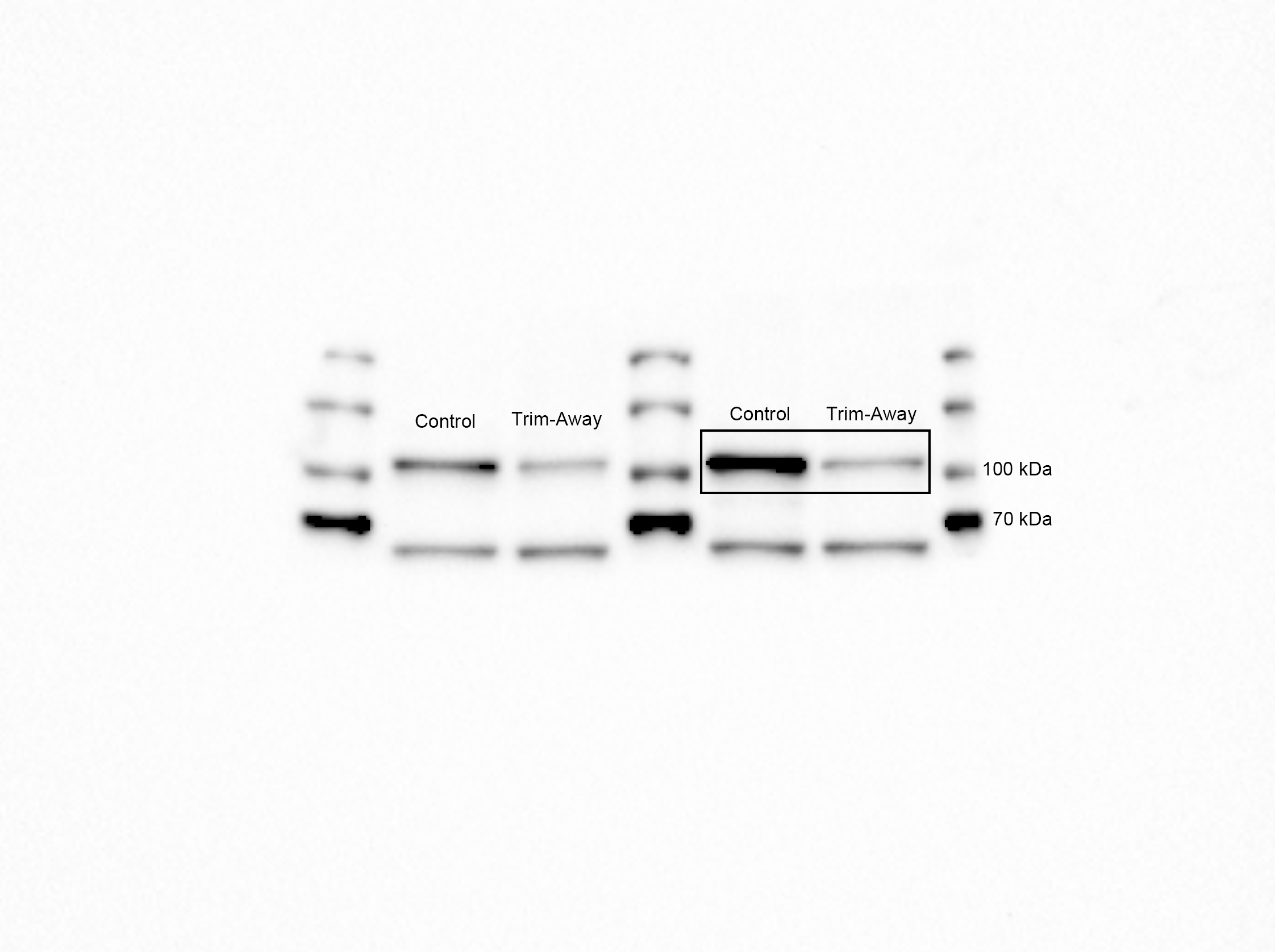

Supplement: Figure 1—source data 2. [file elife-99936-fig1-data2.zip › Figure 1-Source Data 2/Myo19 Trim Away.tif]

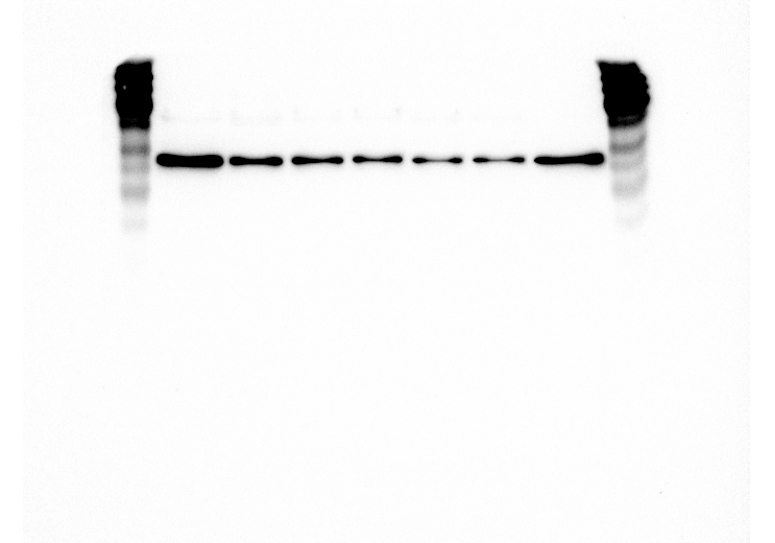

Supplement: Figure 2—source data 1. [file elife-99936-fig2-data1.zip › Figure 2-Source Data 1 (raw)/Fig. 2A_Actin_Bio-Rad 2018-01-19 00hr 28min_Exposure_40.0sec.jpg]

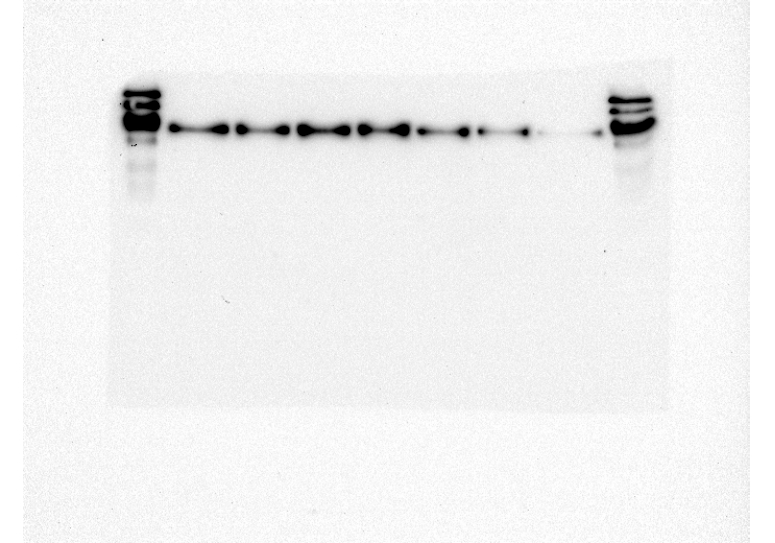

Supplement: Figure 2—source data 1. [file elife-99936-fig2-data1.zip › Figure 2-Source Data 1 (raw)/Fig. 2A_Drp1_Bio-Rad 2018-01-17 19hr 48min_Exposure_360.0sec.jpg]

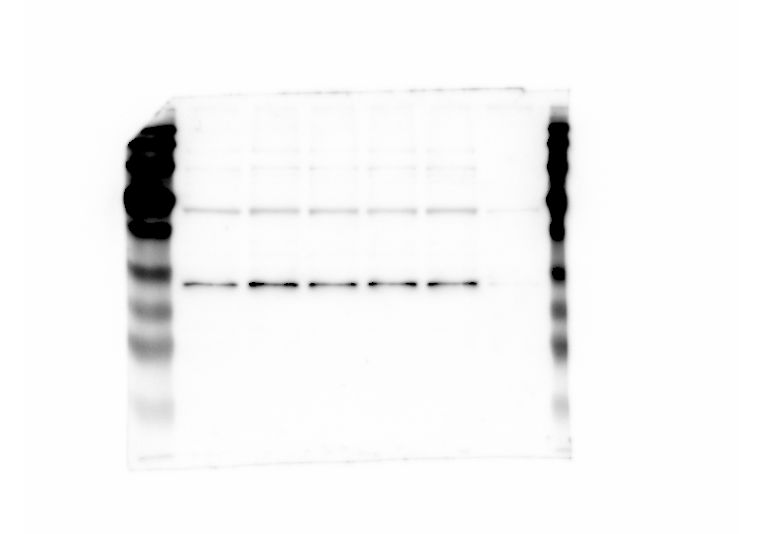

Supplement: Figure 2—source data 1. [file elife-99936-fig2-data1.zip › Figure 2-Source Data 1 (raw)/Fig. 2C_Actin_user 2021-01-24_15h59m41s_Exposure_11.3sec.tif]

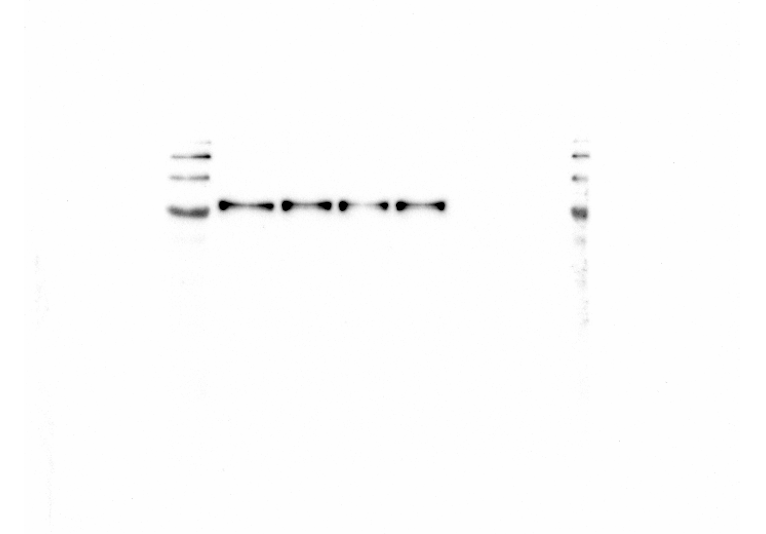

Supplement: Figure 2—source data 1. [file elife-99936-fig2-data1.zip › Figure 2-Source Data 1 (raw)/Fig. 2C_Drp1 user 2021-01-23_18h30m42s_Exposure_120.0sec.tif]

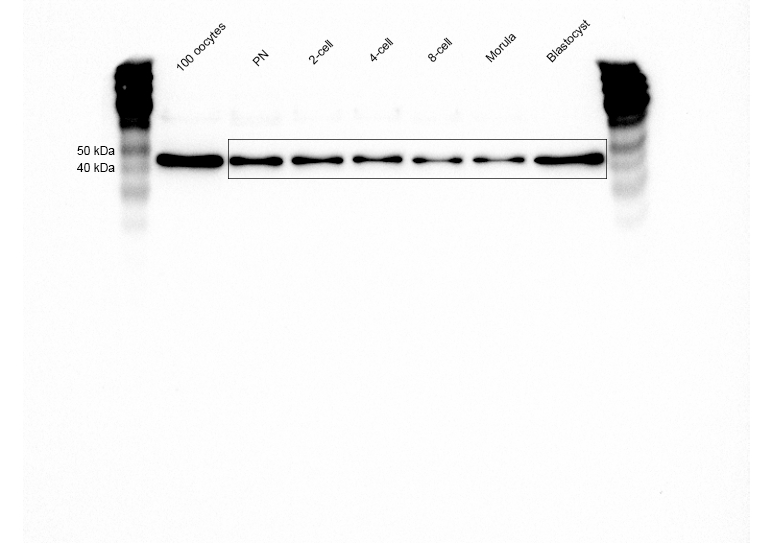

Supplement: Figure 2—source data 2. [file elife-99936-fig2-data2.zip › Figure 2-Source Data 2/Fig. 2A_Actin preimplantation development.tif]

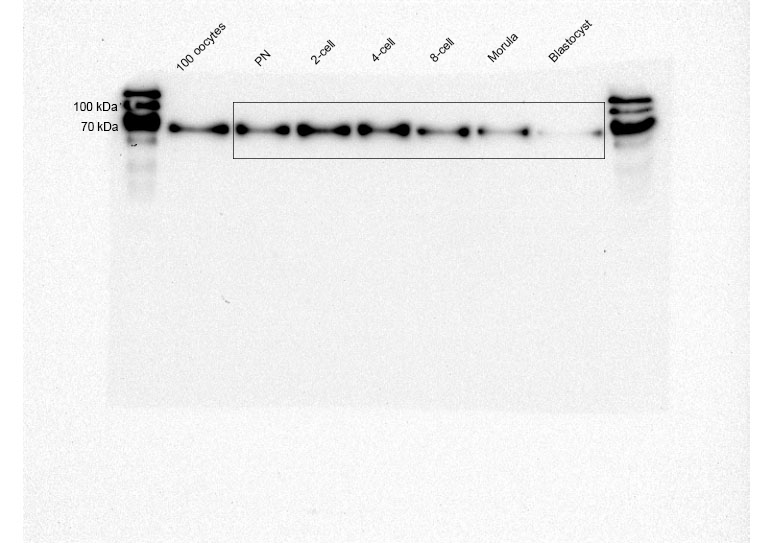

Supplement: Figure 2—source data 2. [file elife-99936-fig2-data2.zip › Figure 2-Source Data 2/Fig. 2A_Drp1 preimplantation development.tif]

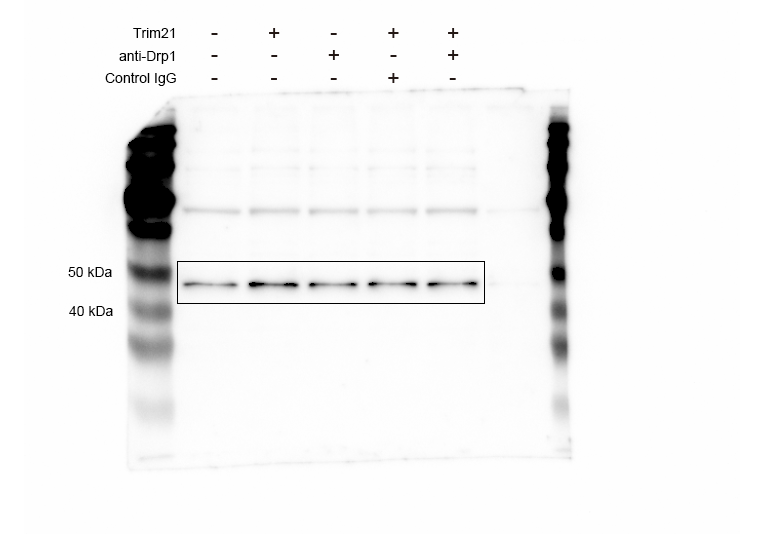

Supplement: Figure 2—source data 2. [file elife-99936-fig2-data2.zip › Figure 2-Source Data 2/Fig. 2C_Actin Trim Away.tif]

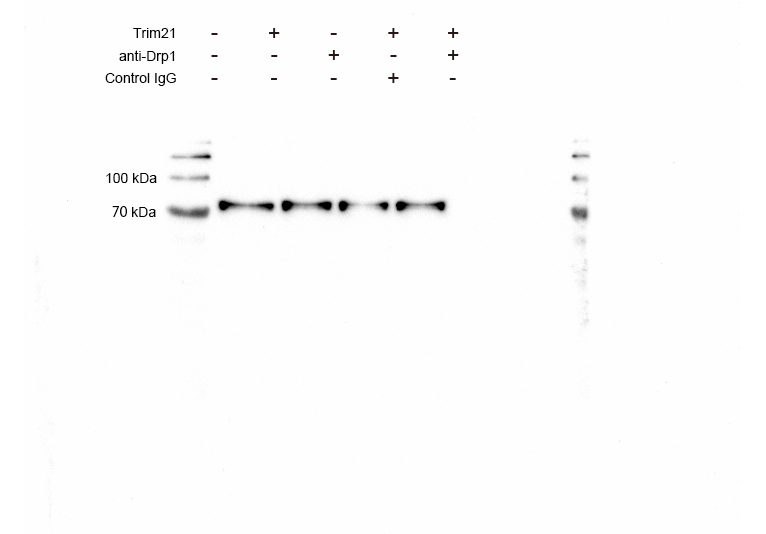

Supplement: Figure 2—source data 2. [file elife-99936-fig2-data2.zip › Figure 2-Source Data 2/Fig. 2C_Drp1 Trim Away.tif]

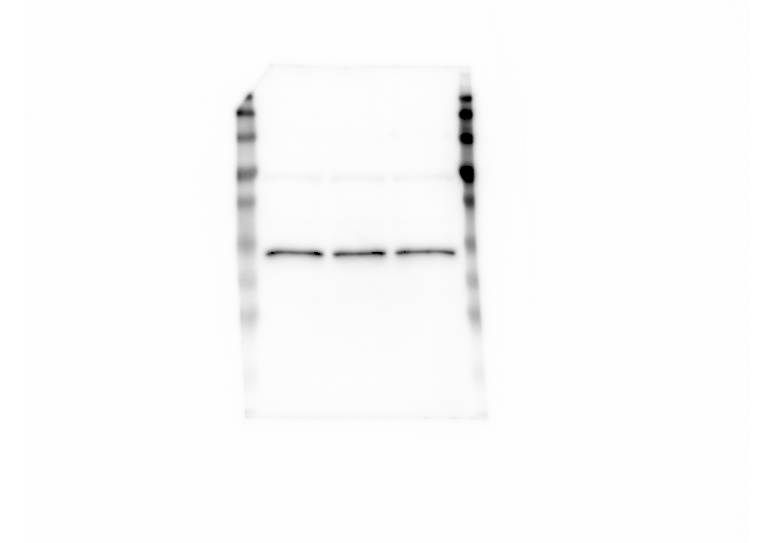

Supplement: Figure 4—source data 1. [file elife-99936-fig4-data1.zip › Figure 4-Source Data 1 (raw)/Actin_Bio-Rad 2020-07-28 14hr 30min_Exposure_1.0sec.tif]

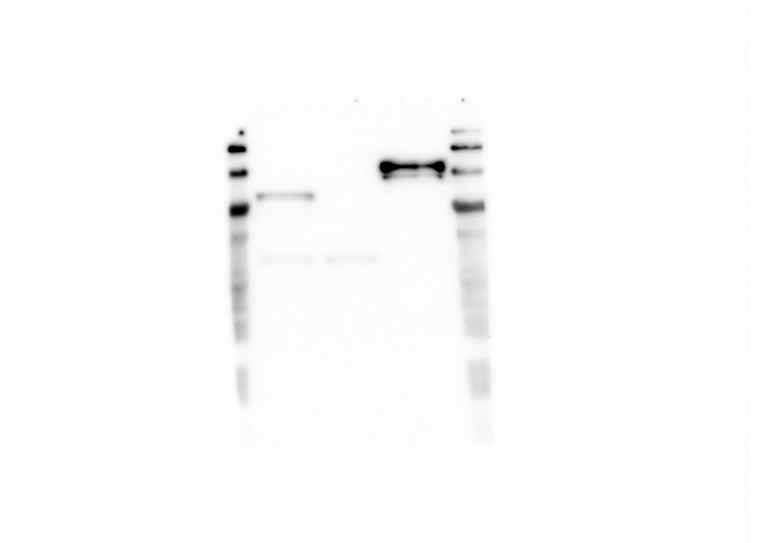

Supplement: Figure 4—source data 1. [file elife-99936-fig4-data1.zip › Figure 4-Source Data 1 (raw)/Drp1_Bio-Rad 2020-07-27 18hr 09min_Exposure_20.0sec.tif]

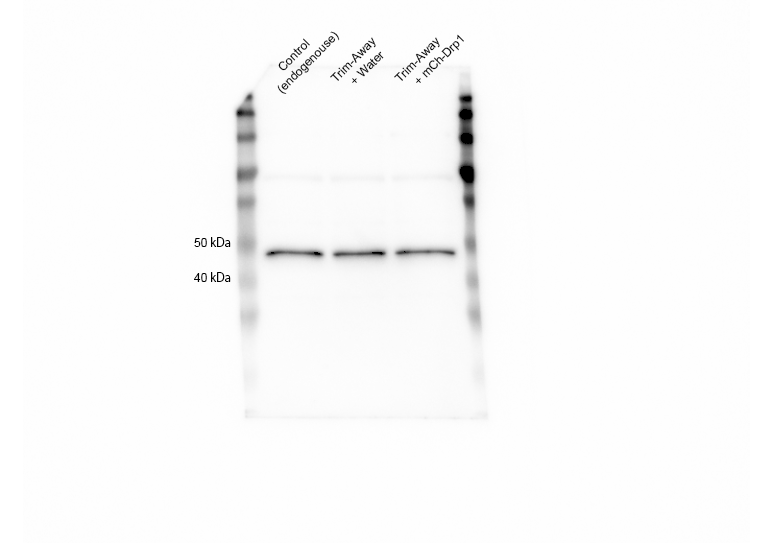

Supplement: Figure 4—source data 2. [file elife-99936-fig4-data2.zip › Figure 4-Source Data 2/Actin expression rescue.tif]

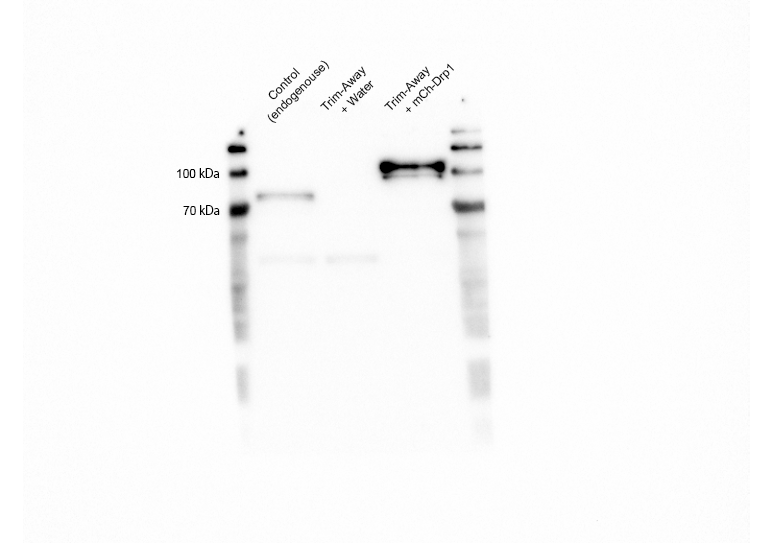

Supplement: Figure 4—source data 2. [file elife-99936-fig4-data2.zip › Figure 4-Source Data 2/Drp1 expression rescue.tif]
